# Undernutrition is alarmingly prevalent in the dysvascular major lower limb amputation population: A prospective cohort study

**DOI:** 10.1371/journal.pone.0342726

**Published:** 2026-09-28

**Authors:** Aniek M. Kolen, Leonie A. Krops, Harriët Jager-Wittenaar, Martijn L. Dijkstra, B. Paul J. A. Keller, Jacques Oskam, Bastiaan P. Vierhout, Jean-Paul P. M. de Vries, Laurens van Walraven, Wim P. Krijnen, Rienk Dekker, Jan H. B. Geertzen

**Affiliations:** 1 Department of Rehabilitation Medicine, University of Groningen, University Medical Center Groningen, Groningen, The Netherlands; 2 Research Group Healthy Ageing, Allied Health Care and Nursing, Hanze University of Applied Sciences, Groningen, The Netherlands; 3 Department of Gastroenterology and Hepatology, Dietetics, Radboud University Medical Center, Nijmegen, The Netherlands; 4 Department Physiotherapy, Human Physiology and Anatomy, Research Unit Experimental Anatomy, Faculty of Physical Education and Physiotherapy, Vrije Universiteit Brussel, Brussels, Belgium; 5 Department of Surgery, Division of Vascular Surgery, University Medical Center Groningen, University of Groningen, Groningen, The Netherlands; 6 Department of Vascular Surgery, Martini Hospital, Groningen, The Netherlands; 7 Department of Surgery, Division of Vascular Surgery, Isala Hospital, Zwolle, The Netherlands; 8 Department of Surgery, Treant Hospital, Emmen, The Netherlands; 9 Department of Vascular Surgery, Medical Center Leeuwarden, Leeuwarden, The Netherlands; University of Michigan, UNITED STATES OF AMERICA

## Abstract

**Introduction:**

People who have undergone dysvascular major lower limb amputation (LLA) are at high risk for adverse clinical outcomes, including undernutrition. However, prevalence of undernutrition in this population is unknown. Therefore, the aim of this study was to determine the nutritional intake and prevalence of undernutrition post-LLA, and to identify determinants of undernutrition.

**Methods:**

In this prospective longitudinal observational study, adults who underwent a dysvascular major LLA were included in six hospitals (n = 81). At several measurement occasions following amputation (<12 days, 5 weeks, 6 months, 9 months), participants’ nutritional intake (24-hour recall) and nutritional status (Patient-Generated Subjective Global Assessment, body mass index, grip strength, mid upper arm circumference, and triceps skinfold) were determined. Furthermore, the Dutch Healthy Diet index was determined. Potential determinants of undernutrition, i.e., age, sex, Charlson comorbidity index, LLA level, uni/bilateral LLA, education level, living situation, smoking status, pain level, and physical activity level, were assessed using mixed effect models.

**Results:**

Median [Q1; Q3] energy intake was 1647 [1178; 2199], 2053 [1516; 2340], 1920 [1263; 2240], and 1797 [1343; 2211] kcal/day (p = 0.002), and protein intake was 76 [59; 100], 88 [68; 114], 84 [64; 101], and 75 [57; 97] grams/day (p = 0.017) at <12 days, 5 weeks, 6 months, and 9 months post-LLA, respectively. Prevalence of undernutrition was 83%, 56%, 28%, and 24%. Determinants of undernutrition were: living in a care facility (OR=6.2, p < 0.001) and lower physical activity (OR=0.96 for every hour of physical activity/week, p = 0.006). Need for nutritional intervention was present in 100%, 82%, 67% and 54% of participants at <12 days, 5 weeks, 6 months, and 9 months post-LLA.

**Conclusion:**

Inadequate nutritional intake and undernutrition are alarmingly prevalent post-LLA. Living in a care facility and lower physical activity are determinants of undernutrition. Interventions to improve nutritional status, to potentially optimize clinical outcomes in the dysvascular major LLA population are highly needed.

## Introduction

People who have undergone a dysvascular major lower limb amputation (LLA) are at high risk for adverse outcomes, as the 30-day mortality rate ranges from 7% to 22% [1], and the 1-year mortality rate is up to 48% [2]. Furthermore, following an LLA, people are at high risk for impaired wound healing [3–5], reduced physical functioning [6], and low quality of life [7–9]. Undernutrition is a potential risk factor for adverse outcomes. Undernutrition is defined as ‘a state resulting from lack of intake or uptake of nutrition that leads to altered body composition (decreased fat free mass) and body cell mass leading to diminished physical and mental function and impaired clinical outcome from disease’ [10].

In a recent scoping review, we concluded that the prevalence of undernutrition in the population of people with dysvascular LLA is unclear, with reported rates ranging widely from 1% to 100% [11]. This variability was mainly attributed to methodological limitations in the included studies and the use of diverse methods to determine undernutrition, such as anthropometry, weight loss, biochemical markers, CT scan analysis, or a combination of these methods [11]. Most studies did not comprehensively capture the nature of undernutrition [10]. Next to this, previous studies were mainly conducted pre- or peri-LLA, and only a limited number of studies were performed post-LLA [11].

Furthermore, qualitative studies involving people at high risk for LLA or post-LLA have identified several factors that affect their diet, e.g., nutrition impact symptoms related to disease and medication (such as pain, nausea, and low appetite), nutritional adaptations for managing comorbidities, financial struggles, ageing, lower physical activity level, and limited nutritional knowledge [12–14]. Currently, to the best of our knowledge, the determinants of undernutrition have not yet been quantitatively investigated in a larger, more generalizable group of people who have undergone a dysvascular major LLA. Moreover, the lack of longitudinal data limits our understanding of how the prevalence of undernutrition and its determinants evolve over time.

Insight into the nutritional intake and the prevalence and determinants of undernutrition in individuals with dysvascular major LLAs is essential to determine whether interventions are needed in the dysvascular major LLA population, and to guide strategies for interventions to prevent or treat undernutrition, aiming to optimize clinical outcomes. Therefore, the aim of this study was to determine the nutritional intake and prevalence of undernutrition at multiple measurement occasions post-LLA, and identify the determinants of undernutrition.

## Methods

This prospective cohort study was conducted in the context of usual care after dysvascular LLA. The study was approved by the Medical Ethical Committee of the University Medical Center Groningen (UMCG; METc UMCG 2022/387), performed in accordance with the Declaration of Helsinki, and reported following STrengthening the Reporting of OBservational studies in Epidemiology (STROBE) [15]. This study was prospectively registered on clinicaltrail.gov [NCT05747066].

### Participants

Inclusion criteria were: having undergone a dysvascular major LLA (i.e., ankle disarticulation, transtibial, transfemoral, knee disarticulation, and hip disarticulation; uni- or bilateral LLA) at minimum a few days and at maximum 7 weeks ago, being 18 years or older, and being physically, mentally, and communicatively able to participate. People were excluded if they had a severe malabsorption disease, or required an ipsilateral reamputation during the study period. Participants were recruited a few days prior or shortly post-LLA from six hospitals in the Northern Netherlands: one academic center, i.e., UMCG, and five non-academic centers: Isala Hospital Zwolle, Martini Hospital Groningen, Medical Center Leeuwarden, Ommelander Hospital Scheemda, and Treant Hospital Emmen. Potential participants were recruited between February 21^st^ and December 18^th^ 2023.

A vascular surgeon or nurse identified potential participants based on inclusion and exclusion criteria. The vascular surgeon, nurse, and/or researcher provided study information and asked whether potential participants were interested in participating. The potential participants had the possibility to ask questions before voluntarily deciding to participate in this study. All participants gave written informed consent prior to participation. Participants were given a €10 voucher for each measurement occasion as a token of appreciation for their time investment.

### Data collection

This study consisted of four measurement occasions: T1 being between two and 12 days post-LLA, T2 at 5 (range 3–7) weeks post-LLA, T3 at 6 (range 5–7) months post-LLA and T4 at 9 (range 8–10) months post-LLA. When T1 was missed, participants could enter the study at T2. The target for the T1 measurement was 4–5 days post‑LLA, but the time window accommodated variation in early postoperative recovery and logistical factors. The T2 measurement was chosen as the time around which sutures were removed, whereby wound healing could be expected. T3 was chosen as the time point at which most people have transferred out of the in-/outpatient rehabilitation facility, and T4 as the time point after which prosthesis use is expected to remain fairly constant.

All measurements were conducted by a researcher (AK) or trained senior year or graduate student (Nutrition and Dietetics or Human Movement Sciences). Data were entered and managed using REDCap [16,17]. Random checks (>10%) were performed to determine input errors in the database.

#### Nutritional intake.

Nutritional intake was assessed by a 24-hour recall, conducted as a face-to-face interview. To standardize this 24-hour recall, a template on information to ask for was used, e.g., product type. Moreover, the book ‘Portion sizes’ [18] was utilized to determine the quantity of consumed food. Nutrition intake logs were used and relatives were asked for guidance when necessary and available. The information from the 24-hour recall was uploaded to Evry software, which used the Dutch Food Composition Database (NEVO-2021), to determine energy and protein intake.

Nutritional intake was determined as absolute and relative intake (kcal or gram/kg actual body weight [19,20]). Body weight was measured at every measurement occasion (Seca 803, SECA UK Ltd). If weight measurement was not possible, the healthcare professional was asked to weigh the participant, or the most recent body weight in the medical record was used. If that was not possible or available, a self-reported body weight was used. If neither was available, body weight was categorized as a missing value, and the body weight from the closest measurement was used to calculate relative intake. If a plaster was worn, 1.5 kg was deducted from the measured body weight, as advised by a certified prosthetist and orthotist. To calculate relative intake, the actual body weight was used without adjusting for the missing limb. This approach was taken because a non-intact body has less metabolically active muscle mass compared to an intact body.

At T1 and T2, relative energy intake ≥30 kcal/kg of actual body weight and relative protein intake ≥1.5 gram/kg of actual body weight were classified as adequate based on international nutritional guidelines for general surgery patients [19,20]. At T3 and T4, protein requirements were still considered slightly increased due to limited anabolic stimuli from low physical activity levels [21–24]. Therefore, an energy intake of ≥25 kcal/kg and a protein intake of ≥1.2 gram/kg were classified as adequate at T3 and T4 [20].

Diet quality was evaluated by the Dutch Healthy Diet index 2015 (DHD), based on the data obtained from the 24-hour recall. The 15 components of the DHD represent the 15 food-based Dutch dietary guidelines of 2015 [25]. Possible scores range from 0 to 10 for each component, leading to a total DHD score between 0 (no adherence) and 150 (complete adherence to guidelines) [26]. The DHD was not calculated if participants consumed less than 1500 kcal per day from regular food products (excluding nutritional supplements, tube feeding, and/or nutrition by vein), as the score is likely not valid with a low intake. Furthermore, participants would likely not meet their micronutrient requirements [27]. In cases where participants were not in control of their meal preparations, e.g., during hospitalization, and no information was available from cooking staff or relatives, we calculated nutritional content using 10 grams of liquid cooking fat and 1 gram of salt per 100 grams of product, if applicable.

#### Nutritional status.

Undernutrition was determined using the Patient-Generated Subjective Global Assessment (PG-SGA, Dutch version [28]). The patient-completed component (PG-SGA Short Form), consists of four boxes addressing: (1) weight loss over the past 2 weeks, 1 month, and 6 months; (2) changes in food intake in the past month; (3) nutrition impact symptoms in the past two weeks; and (4) activities and function in the past month [28,29]. The researcher completed the second part of the PG‑SGA, including scoring of weight loss, disease burden and metabolic demand, and physical examination [28,29].

Body weight post-LLA was adjusted for the missing limb in case of major LLA, based on the calculations of Durkin [30] and expert opinion estimates for different amputation levels [31,32].

Based on Worksheet 5, participants were classified as Stage A (well-nourished), Stage B (moderate or suspected undernutrition), or Stage C (severely undernourished). For analyses, undernutrition was dichotomized as stage A versus stage B/C due to the small number of participants in Stage C, consistent with previous studies [33–36]. The PG-SGA numerical score (0 - > 52) was calculated, to determine nutritional triage recommendations: 0–1 No intervention required at this time. Re-assessment on routine and regular basis during treatment; 2–3 Patient & family education by dietitian, nurse, or other clinician with pharmacologic intervention as indicated by symptom survey and lab values as appropriate; 4–8 Requires intervention by dietitian, in conjunction with nurse or physician as indicated by symptoms; A score of ≥9 Indicates a critical need for improved symptom management and/or nutrient intervention options [29].

Additionally, muscle mass (mid upper arm circumference and triceps skinfold), hand grip strength, and body mass index (BMI) were evaluated. Muscle mass was estimated through the measurement of the mid upper arm circumference (Seca 201 circumference measuring tape, SECA UK Ltd) and the triceps skinfold (Harpenden, Holtain Ltd., Crosswell, UK), conducted at the midpoint between the acromion and the olecranon of the dominant arm. Muscle mass was calculated as: mid upper arm circumference – (triceps skinfold x π). Data were excluded if deemed unreliable, e.g., when measurements were difficult to perform due to the participant’s position, excess skin tissue affecting accuracy, or other factors compromising reliability.

Grip strength was determined using a hand dynamometer (Jamar, TEC Inc., Clifton, NJ, USA). The participant was in a seated position with the elbow 90 degrees flexed, without the elbow making contact with any surface or the body. Grip strength of the dominant hand was measured three times, of which the highest grip strength was used for analysis. The researcher provided verbal encouragement during the measurement. Grip strength was categorized as reduced grip strength (<30 kg for males and <20 kg for females), or sufficient grip strength (≥30 kg for males and ≥20 kg for females) [37].

BMI (adjusted for LLA) was assessed by height, body weight, level of LLA, sex, and age. Height was measured, when possible, with a Seca 201 measuring tape (SECA UK Ltd) while participants stood upright against a wall. If the participant was not able to stand up or had a bilateral LLA, self-reported height (before LLA) was used. BMI was adjusted for LLA to compare to BMI cutoffs for people with an intact body, and categorized as low (<20 kg/m^2^ for people younger than 70 years, or <22 kg/m^2^ for people of 70 years or older) or not low (≥20 kg/m^2^ for people younger than 70 years, or ≥22 kg/m^2^ for people of 70 years or older) [38]. Additionally, a BMI of 25–29.9 kg/m^2^ was categorized as overweight and a BMI ≥ 30 kg/m^2^ was classified as obese.

Measurement instruments, i.e., skinfold caliper and hand dynamometer, were calibrated yearly.

#### Participants’ characteristics and possible determinants.

Participants’ characteristics were obtained by a self-reported questionnaire including: age at moment of LLA, sex (male, female), characteristics of LLA (uni- or bilateral, level(s) of LLA), previous history of revascularization and/or LLA, comorbidities (self-reported Charlson Comorbidity Index (CCI) [39]), average and worst pain in the last week at every measurement occasion (Visual Analogue Scale (VAS)), living situation at every measurement occasion (type of facility or home, alone or with others), education level (categorized as low: elementary or high school; moderate: vocational college; high: (applied) university), smoking status (never, previous, current), mobility pre-LLA (independent outdoor ambulator, independent indoor ambulator, aid depended, wheelchair or bedridden), K-level, adherence to Dutch physical activity guidelines at every measurement occasion (yes, no) [40], and hours per week being physically active based on the adapted-SQUASH at every measurement occasion [41]. Lastly, we assessed whether the participant received dietary counselling in the three months preceding the LLA, during hospital admission for LLA, and in the time between measurements (yes/no), respectively.

### Statistical analysis

Descriptive statistics were used to summarize participant characteristics, nutritional intake, and nutritional status at every measurement occasion and between measurement occasions. Continuous variables were presented as mean and standard deviation, or as median and interquartile range, depending on normality of the underlying distribution. Categorical variables were presented as frequencies and percentages.

To investigate changes over time, descriptive and testing analyses were used. Descriptive analyses were used to determine the number of participants improving or worsening PG-SGA category over time. Furthermore, to investigate the change over time for nutritional intake (absolute and relative intake, and diet quality), PG-SGA numerical score, grip strength, muscle mass, body weight, and BMI, a mixed model analysis with the restricted maximum-likelihood method and an unstructured correlation structure was used, using measurement occasion as time factor, with a random intercept for participants to account for individual variability. Moreover, analysis was corrected for age, sex, amputation level (low, high), education level (low, mod/high), CCI score without age, smoker (yes, previous/never), and physical activity in hours per week. A univariate mixed effects logistic regression analysis was conducted to investigate the determinants of undernutrition (PG-SGA category A vs B/C). Multivariate mixed effect logistic regression analysis failed to converge.

Significance of fixed effects of measurement occasion was tested by likelihood ratio, where the results were considered statistically significant at a p-value <0.05. The validity of the model estimation was investigated by an analysis of the residuals including Cook’s distance. Analysis was performed in the statistical programming language R (v4.4.0) [42], using the lmer function of the lme4 package [43]. Furthermore, to evaluate pairwise differences between measurement occasions, planned linear contrasts were used with the general linear hypothesis testing framework in the multcomp package and computed adjusted p-values [44,45]. No imputation was performed for missing data, since mixed model analyses are flexible for handling missing data, and research has shown no obvious gain for multiple imputations in mixed model analyses [46]. The reported β coefficients reflect estimates obtained after incorporating potential covariates into the model.

## Results

In total, 81 participants were included and the participation rate of potential participants was 76% (Fig 1). The average age at the moment of LLA was 69.7 ± 10.5 years, and 59 participants (73%) were male. Most participants had a unilateral transtibial amputation. Moreover, 37% of participants had a dietitian consultation pre-LLA, 38% during hospital admission, 53% post-hospital discharge to T2, 40% between T2-T3, and 29% between T3-T4 (Table 1). T1, T2, T3 and T4 were performed 4.7 ± 1.8 days, 5.1 ± 0.6 weeks, 5.9 ± 0.3 months, and 9.0 ± 0.3 months post-LLA, respectively.

**Table 1:** Characteristics of participants (n = 81).

		Mean ± SD, median [Q1; Q3], or n (%)
Age (years)		69.7 ± 10.5
Sex (male)		59 (73%)
Amputation level		
	Transtibial	59 (73%)
	Knee disarticulation	3 (4%)
	Transfemoral	19 (24%)
Bilateral amputation		13 (16%)
	Transtibial	10
	Transfemoral	3
Living situation pre-LLA		
	Home	75 (93%)
	Alone	24
	Care facility	6 (7%)
Living situation (T2; T3; T4)		
	Home	22 (31%); 45 (78%); 48 (87%)
	Skilled nursing facility	41 (58%); 3 (5%); 0 (0%)
	Rehabilitation facility	1 (1%); 0 (0%); 0 (0%)
	Nursing home	3 (4%); 9 (16%); 6 (6%)
	Admitted in hospital	2 (3%); 0 (0%); 0 (0%)
	Other	2 (3%); 1 (2%); 1 (2%)
Educational level		
	Low	44 (54%)
	Moderate	22 (27%)
	High	14 (17%)
	Unknown	1 (1%)
Smoking status		
	Current	23 (28%)
	Previous	37 (46%)
	Never	21 (26%)
Mobility pre-LLA		
	Independent outdoor ambulator	24 (30%)
	Independent indoor ambulator	14 (17%)
	Aid depended	38 (47%)
	Wheelchair or bedridden	5 (6%)
K-level (T3; T4)		
	0	30 (52%); 30 (55%)
	1	8 (14%); 8 (15%)
	2	17 (29%); 11 (20%)
	3	3 (5%); 6 (11%)
	4	0 (0%); 0 (0%)
Meeting Dutch physical activity guidelines (yes) (T1; T2; T3; T4)		12 (16%); 39 (55%); 27 (47%); 15 (27%)
Physical activity (min/week)		240 [90; 600]; 525 [245; 880]; 678 [344; 1176]; 885 [525; 1733]
Pain average (VAS 0–10) (T1; T2; T3; T4)		5.4 [2.3; 7.6]; 1.7 [0.0; 4.8]; 0.8 [0.0; 4.8]; 1.8 [0.0; 5.3]
Pain worst (VAS 0–10) (T1; T2; T3; T4)		9.7 [7.3; 10]; 4.6 [0.0; 8.6]; 2.5 [0.0; 7.2]; 4.8 [0.0; 8.1]
Charlson Comorbidity Index		6.1 ± 2.3
Charlson Comorbidity Index (without age)		3.6 ± 2.0
Diabetes		50 (62%)
Previous minor/major LLAs		0 [0; 1]
Previous vascular surgeries		2 [1; 4]
Dietitian consult (yes)		
	During 3 months pre-amputation	30 (37%)
	During hospital admission	31 (38%)
	Between post-hospital admission-T2	38 (53%)
	Between T2-T3	23 (40%)
	Between T3-T4	16 (29%)

Abbreviations. SD, standard deviation; VAS, Visual Analogue Scale; LLA, lower limb amputation.

The total sample size was 81. The number of participants at T1, T2, T3, and T4 was 76, 71, 58, and 55, respectively. Age at moment of LLA.

Educational level: low: elementary or high school; moderate: vocational college; high: (applied) university.

**Fig 1:**
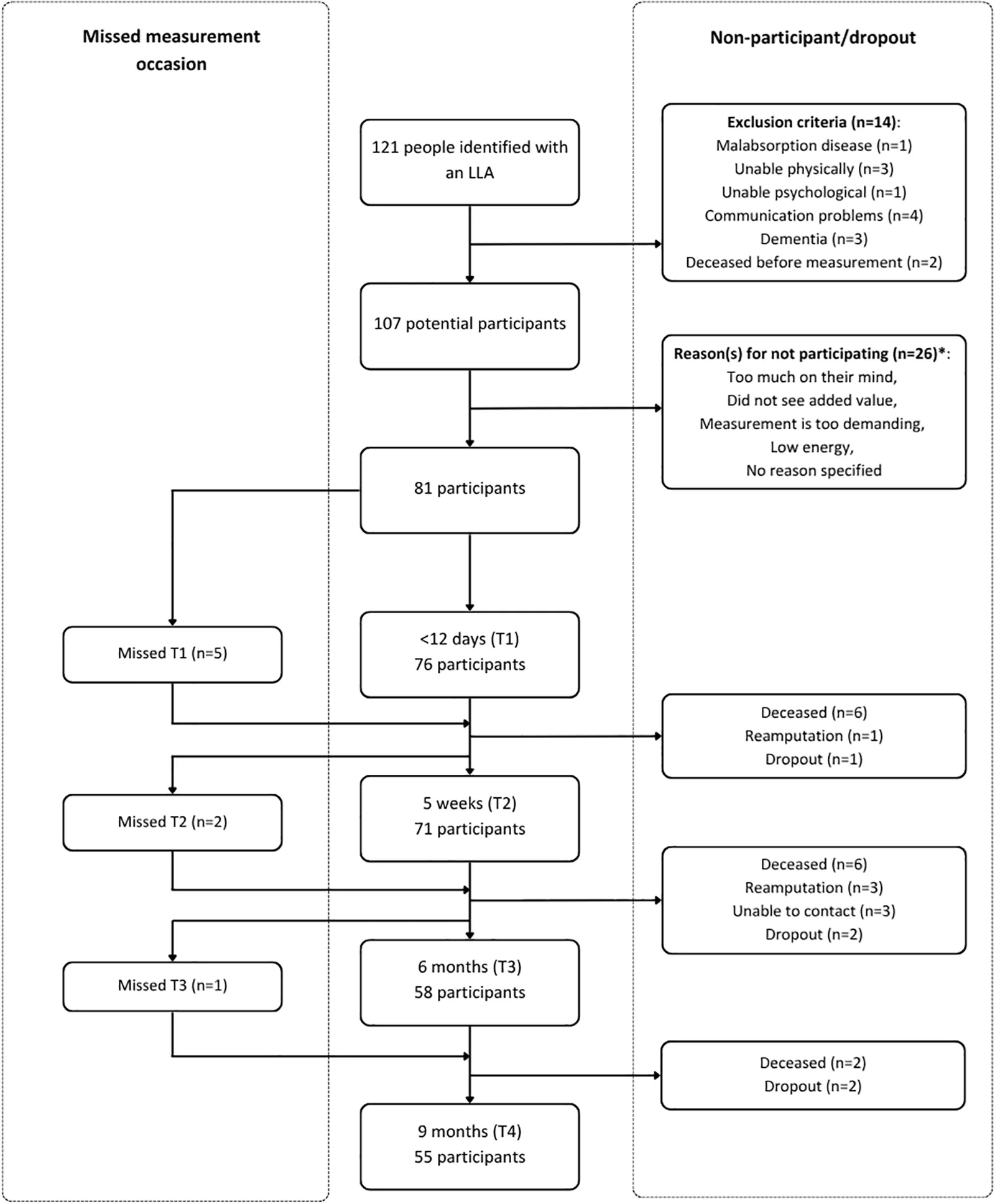
Illustration of recruitment, displaying the number of people identified, potential participants, enrolled participants, and number of participants per measurement occasion. *One or more reasons per potential participant for not being willing to participate.

### Nutritional intake

Nutritional intake was unknown for 4% of measurements, due to memory problems of the participant and fatigue/time constraints. Median absolute and relative energy intake ranged from 1647 to 2053 kcal/day and 21.4 to 26.3 kcal/kg/day. Median protein intake ranged from 75 to 88 gram/day and 0.9 to 1.2 gram/kg/day across the measurement occasions. The DHD index ranged from 66 to 71 across measurement occasions, with the lowest scores in the components of legumes, nuts, and fish (Table 2).

**Table 2:** Nutritional intake at various measurement occasions post-amputation as median [Q1; Q3].

		T1 <12 days	T2 5 weeks	T3 6 months	T4 9 months	p-value^a^	p-value^b^
**Energy**		n = 70	n = 70	n = 56	n = 54		
	Kcal/day	1647 [1178; 2199]	2053 [1516; 2340]	1920 [1263; 2240]	1797 [1343; 2211]	0.002^†^	0.002^†‡¥^
	Kcal/kg/day^c^	21.5 [14.5; 28.3]	26.3 [20.0; 33.0]	21.4 [15.6; 31.4]	21.6 [17.0; 27.1]	<0.001^†‡¥^	<0.001^†‡¥^
	≥25 kcal/kg/day	27 (39%)	38 (54%)	21 (39%)	16 (30%)		
	≥30 kcal/kg/day	14 (20%)	28 (40%)	17 (30%)	12 (22%)		
**Protein**							
	Gram/day	76 [59; 100]	88 [68; 114]	84 [64; 101]	75 [57; 97]	0.048	0.017^¥^
	Gram/kg/day^c^	1.0 [0.7; 1.3]	1.2 [0.9; 1.6]	1.0 [0.8; 1.4]	0.9 [0.7; 1.2]	0.004^†‡¥^	0.001^†‡ ¥^
	≥1.2 gram/kg/day	23 (33%)	35 (50%)	22 (39%)	13 (24%)		
	≥1.5 gram/kg/day	11 (16%)	24 (34%)	10 (18%)	5 (9%)		
**Diet quality**		n = 38	n = 47	n = 37	n = 34		
	DHD index (0–150)^d^	65.7 ± 15.9	66.5 ± 11.2	69.5 ± 15.5	70.5 ± 17.3	0.209	0.381
	Vegetables	3.7 [1.7; 6.5]	4.5 [2.5; 7.5]	4.5 [2.3; 7.5]	6.1 [4.5; 10]	0.045^#^	0.027^#^
	Fruit	3.6 [0.0; 6.5]	2.5 [0.0; 8.3]	0.0 [0.0; 6.8]	0.0 [0.0; 6.3]	0.395	0.139
	Wholegrain products	5.4 [4.0; 7.8]	5.0 [1.8; 7.7]	4.7 [0.0; 8.9]	5.8 [3.9; 10]	0.332	0.312
	Legumes	0.0 [0.0; 0.0]	0.0 [0.0; 0.0]	0.0 [0.0; 0.0]	0.0 [0.0; 0.0]	0.097	0.098
	Nuts	0.0 [0.0; 0.0]	0.0 [0.0; 0.0]	0.0 [0.0; 0.0]	0.0 [0.0; 0.0]	0.613	0.535
	Dairy	1.3 [0.0; 6.6]	6.2 [0.0; 8.3]	3.2 [0.5; 8.5]	2.8 [0.2; 9.0]	0.470	0.607
	Fish	0.0 [0.0; 0.0]	0.0 [0.0; 0.0]	0.0 [0.0; 0.0]	0.0 [0.0; 0.0]	0.374	0.613
	Tea	0.0 [0.0; 2.5]	0.0 [0.0; 5.6]	0.0 [0.0; 4.4]	0.0 [0.0; 5.0]	0.864	0.914
	Fats and oils	0.0 [0.0; 0.0]	0.0 [0.0; 6.3]	10 [0.0; 10]	10 [0.0; 10]	<0.001^§#‡¥^	<0.001^§#‡¥^
	Coffee	10 [10; 10]	10 [10; 10]	10 [10; 10]	10 [10; 10]	ND	ND
	Red meat	10 [10; 10]	10 [10; 10]	10 [10; 10]	10 [10; 10]	0.090	0.043^†^
	Processed meat	0.0 [0.0; 6.0]	0.0 [0.0; 0.0]	0.0 [0.0; 4.0]	0.0 [0.0; 6.8]	0.309	0.263
	Sweet beverages and fruit juices	10 [1.0; 10]	10 [0.0; 10]	4.0 [0.0; 10]	10 [0.0; 10]	0.613	0.477
	Alcohol	10 [10; 10]	10 [10; 10]	10 [10; 10]	10 [8.4; 10]	<0.001^#¥¶^	<0.001^#¥¶^
	Salt	6.6 [3.5; 9.9]	6.0 [1.7; 8.6]	5.2 [2.6; 8.1]	5.7 [2.0; 9.2]	0.546	0.629

Abbreviations. Kcal, kilocalorie; kg, kilogram; DHD, Dutch Healthy Diet; ND, not defined.

a  = Analysed by mixed effect model, incorporating time.

b  = Analysed by mixed effect model, incorporating time, age, sex, amputation level (low, high), education level (low, mod/high), CCI score without age, smoker (yes, previous/never), and physical activity in hours per week.

c  = Body weight was unknown for 1, 2, 4, and 7 participants at T1, T2, T3, and T4, respectively. Relative nutritional intake was calculated using the actual body weight from the closest measurement occasion.

d  = Each component of the Dutch Healthy Diet index is a score between 0 and 10. A higher score indicates more compliance with the Dutch dietary guidelines of 2015 [25,26].

† Significant difference between T1 and T2 (p < 0.05).

§ Significant difference between T1 and T3 (p < 0.05).

# Significant difference between T1 and T4 (p < 0.05).

‡ Significant difference between T2 and T3 (p < 0.05).

¥ Significant difference between T2 and T4 (p < 0.05).

¶ Significant difference between T3 and T4 (p < 0.05).

Cooks’s distance ranged from 0.06 to 0.15 for energy intake, 0.07 to 0.16 for protein intake, and 0.04 to 0.99 for diet quality.

Absolute energy intake (p = 0.002), relative energy intake (p < 0.001), absolute protein intake (p = 0.017), and relative protein intake (p = 0.001) significantly differed across the measurement occasions, whereas DHD index (p = 0.381) did not. Absolute and relative energy intake and relative protein intake were significantly higher at T2 compared to T1, T3, and T4, while absolute protein intake was only significantly higher at T2 compared to T4, and not significant between T2 and T3 (β = 14 gram/day, p = 0.064, Table 2).

### Nutritional status

At T1, T2, T3, and T4, respectively 38%, 8%, 7%, and 4% of the participants were severely undernourished and 45%, 48%, 21%, and 20% of the participants had moderate or suspected undernutrition. In the univariate analysis, the prevalence of undernutrition (dichotomized) significantly decreased over time (p < 0.001), with a significant decrease between T1 and T2 (β = 1.69, p < 0.001), T1 and T3 (β = 3.30, p < 0.001), T1 and T4 (β = 3.57, p < 0.001), T2 and T3 (β = 1.61, p = 0.003), and T2 and T4 (β = 1.88, p < 0.001). However, no significant difference between T3 and T4 (β = 0.27, p = 0.948).

Between measurement occasions T1 and T2, the nutritional status remained unchanged in 26 participants (39%), showed improvement in 32 (48%), and deteriorated in eight participants (12%). Between T2 and T3, nutritional status remained the same in 30 participants (53%), improved in 20 participants (35%), and deteriorated in seven participants (12%). Between T3 and T4, nutritional status remained the same in 36 participants (67%), improved in 11 (20%), and deteriorated in seven participants (13%, Fig 2).

**Fig 2:**
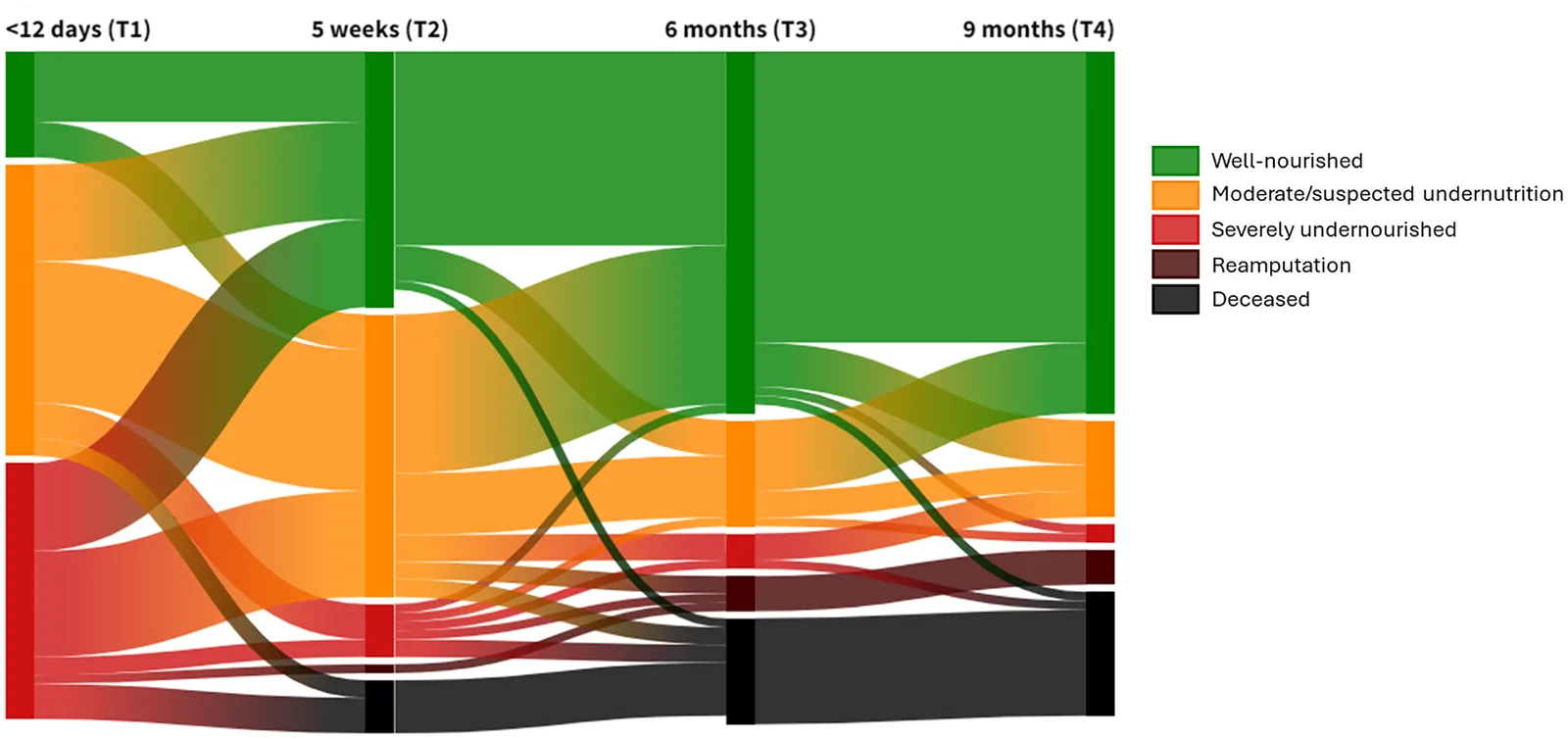
Prevalences of undernutrition according to PG-SGA categories (well-nourished, moderate or suspected undernutrition, severely undernourished) at various measurement occasions post-amputation. A single participant did not complete T2, and deceased before T3.

Table 3 shows the median PG-SGA numerical scores per measurement occasion, as well as the separate results per box/worksheet. The median PG-SGA scores changed over time, reaching the highest median value of 14 [11; 19] at T1, which decreased to 8 [5; 11], 5 [3; 7], and 4 [2; 6] at T2, T3, and T4, respectively (p < 0.001). PG-SGA scores significantly differed between the measurement occasions, except between T3 and T4 (β = −0.4, p = 0.920). The need for nutritional intervention ranged from 100−54% between T1 and T4. At T1, 40% of participants had lost ≥5% of their body weight in the last month, of whom 15% had lost ≥10%. Nutrition impact symptoms were present in 72%, 45%, 31%, and 25% of participants at T1, T2, T3, and T4, of which the following symptoms were most prevalent: no appetite, nausea, vomiting, early satiation, funny/no taste, and did not like the provided food (mentioned under other symptoms, Table 3, Fig 3).

**Table 3:** Distribution of PG-SGA numerical scores at various measurement occasions post-amputation (median [Q1; Q3]) with p-values from mixed model testing without and with correcting for confounding variables.

		T1 (n = 76)	T2 (n = 71)	T3 (n = 58)	T4 (n = 55)	p-value^a^	p-value^b^
		<12 days	5 weeks	6 months	9 months		
**PG-SGA total score**		**14 [11; 19]**	**8 [5; 11]**	**5 [3; 7]**	**4 [2; 6]**	<0.001^†§#‡¥^	<0.001^†§#‡¥^
	Well-nourished	7 [6; 9]	7 [4; 8]	4 [2; 6]	3 [2; 4]		
	Moderate/suspected undernutrition	14 [11; 16]	9 [6; 11]	7 [5; 9]	8 [7; 14]		
	Severely undernourished	19 [16; 24]	18 [14; 24]	18 [13; 20]	17 [12;21]		
**Nutritional triage recommendations** ^**c**^							
≥9 points		67 (87%)	28 (39%)	10 (17%)	8 (15%)		
4-8 points		10 (13%)	37 (52%)	29 (50%)	21 (38%)		
2-3 points		0 (0%)	5 (7%)	16 (28%)	22 (40%)		
0-1 points		0 (0%)	1 (1%)	3 (5%)	4 (7%)		
**Results per box/worksheet**							
**Box 1: Weight history**		**2 [0; 4]**	**0 [0; 2]**	**0 [0; 0]**	**0 [0; 0]**	<0.001^†§#^	<0.001^†§#^
Weight loss last month							
	≥10%	11 (15%)	1 (1%)	1 (2%)	1 (2%)		
	5-9.9%	19 (25%)	6 (9%)	1 (2%)	2 (4%)		
	3-4.9%	6 (8%)	11 (16%)	6 (10%)	4 (7%)		
	2-2.9%	3 (4%)	12 (17%)	2 (3%)	0 (0%)		
	0-1.9%	36 (47%)	40 (56%)	44 (76%)	42 (76%)		
	Missing	1 (1%)	1 (1%)	4 (8%)	6 (11%)		
Weight decreased last 2 weeks (yes)		45 (59%)	14 (20%)	4 (7%)	5 (9%)		
Missing		0 (0%)	0 (0%)	1 (2%)	2 (4%)		
**Box 2: Food intake**		**1 [0; 1]**	**0 [0; 1]**	**0 [0; 0]**	**0 [0; 0]**	<0.001^†§#^	<0.001^†§#^
Food intake during the past month							
	Less than usual	48 (63%)	27 (38%)	13 (22%)	11 (20%)		
	Unchanged	26 (34%)	32 (45%)	39 (67%)	39 (71%)		
	More than usual	2 (3%)	12 (17%)	6 (10%)	5 (9%)		
Current type of food							
	Normal food but less amount	29 (38%)	24 (34%)	11 (19%)	10 (17%)		
	Little solid food	15 (20%)	3 (4%)	3 (5%)	1 (2%)		
	Only liquids	2 (3%)	0 (0%)	0 (0%)	0 (0%)		
	Only nutritional supplements	0 (0%)	0 (0%)	0 (0%)	0 (0%)		
	Very little of anything	2 (3%)	0 (0%)	0 (0%)	2 (4%)		
	Only tube feeding or only nutrition by the vein	1 (1%)	0 (0%)	0 (0%)	0 (0%)		
	Not applicable	27 (36%)	43 (61%)	44 (76%)	42 (76%)		
**Box 3: Symptoms** ^**d**^		**4 [1; 7]**	**0 [0; 3]**	**0 [0; 1]**	**0 [0; 1]**	<0.001^†§#^	<0.001^†§#^
	No problems eating	21 (28%)	39 (55%)	40 (69%)	41 (75%)		
	No appetite, just did not feel like eating	40 (53%)	16 (23%)	9 (16%)	10 (18%)		
	Nausea	16 (21%)	5 (7%)	2 (3%)	3 (6%)		
	Constipation	9 (12%)	1 (1%)	0 (0%)	0 (0%)		
	Mouth sores	4 (5%)	0 (0%)	1 (2%)	0 (0%)		
	Things taste funny or have no taste	13 (17%)	3 (4%)	1 (2%)	2 (4%)		
	Problems swallowing	6 (8%)	0 (0%)	0 (0%)	0 (0%)		
	Vomiting	12 (16%)	4 (6%)	1 (2%)	0 (0%)		
	Diarrhea	8 (11%)	3 (4%)	0 (0%)	0 (0%)		
	Dry mouth	10 (13%)	2 (3%)	0 (0%)	0 (0%)		
	Smells bother me	4 (5%)	0 (0%)	1 (2%)	0 (0%)		
	Feel full quickly	10 (13%)	10 (14%)	2 (3%)	1 (2%)		
	Fatigue	16 (21%)	5 (7%)	2 (3%)	3 (6%)		
	Pain	13 (17%)	4 (6%)	1 (2%)	1 (2%)		
	Other^e^	19 (25%)	14 (20%)	9 (16%)	5 (9%)		
**Box 4: Activities and function**		**3 [2; 3]**	**2 [1; 3]**	**1 [1; 2]**	**1 [1; 2]**	<0.001^§#‡ ¥^	<0.001^§#‡ ¥^
Self-rated activity level							
	Normal with no limitations	6 (8%)	0 (0%)	3 (5%)	6 (11%)		
	Not my normal self, able to be up and about with normal activities	7 (9%)	19 (27%)	27 (47%)	29 (53%)		
	Not feeling up to most things, but in bed or chair less than half day	13 (17%)	21 (30%)	19 (33%)	15 (27%)		
	Able to do little activity and spend most of the day in bed or chair	38 (50%)	28 (39%)	8 (14%)	5 (9%)		
	Pretty much bed ridden, rarely out of bed	12 (16%)	3 (4%)	1 (2%)	0 (0%)		
**Worksheets**		**5 [4; 6]**	**3 [2; 4]**	**2 [1; 4]**	**2 [1; 3]**	<0.001^†§#‡¥^	<0.001^†§#‡¥^

Abbreviations. PG-SGA, Patient-Generated Subjective Global Assessment.

a  = Analysed by mixed effect model, incorporating time.

b  = Analysed by mixed effect model, incorporating time, age, sex, amputation level (low, high), education level (low, mod/high), CCI score without age, smoker (yes, previous/never), and physical activity in hours per week.

c  = Triage based on PG-SGA numerical score: 0–1 No intervention required at this time. Re-assessment on routine and regular basis during treatment. 2–3 Patient & family education by dietitian, nurse, or other clinician with pharmacologic intervention as indicated by symptom survey and lab values as appropriate. 4–8 Requires intervention by dietitian, in conjunction with nurse or physician as indicated by symptoms. ≥ 9 Indicates a critical need for improved symptom management and/or nutrient intervention options.

d  = Multiple symptoms can be reported. Therefore, the total is not 100%.

e  = Other symptoms included: dislike of the taste, texture, and/or smell of the provided food, limited or no taste and/or smell due to COVID, eating alone, difficulties preparing food, psychological burden or stress, shortness of breath, reflux, unpleasant feeling in the tongue, side effects of medication, low physical activity level, and a new denture with a plastic taste.

† Significant difference between T1 and T2 (p < 0.05).

§ Significant difference between T1 and T3 (p < 0.05).

# Significant difference between T1 and T4 (p < 0.05).

‡ Significant difference between T2 and T3 (p < 0.05).

¥ Significant difference between T2 and T4 (p < 0.05).

¶ Significant difference between T3 and T4 (p < 0.05).

Cook’s distance ranged from 0.05 to 0.28.

**Fig 3:**
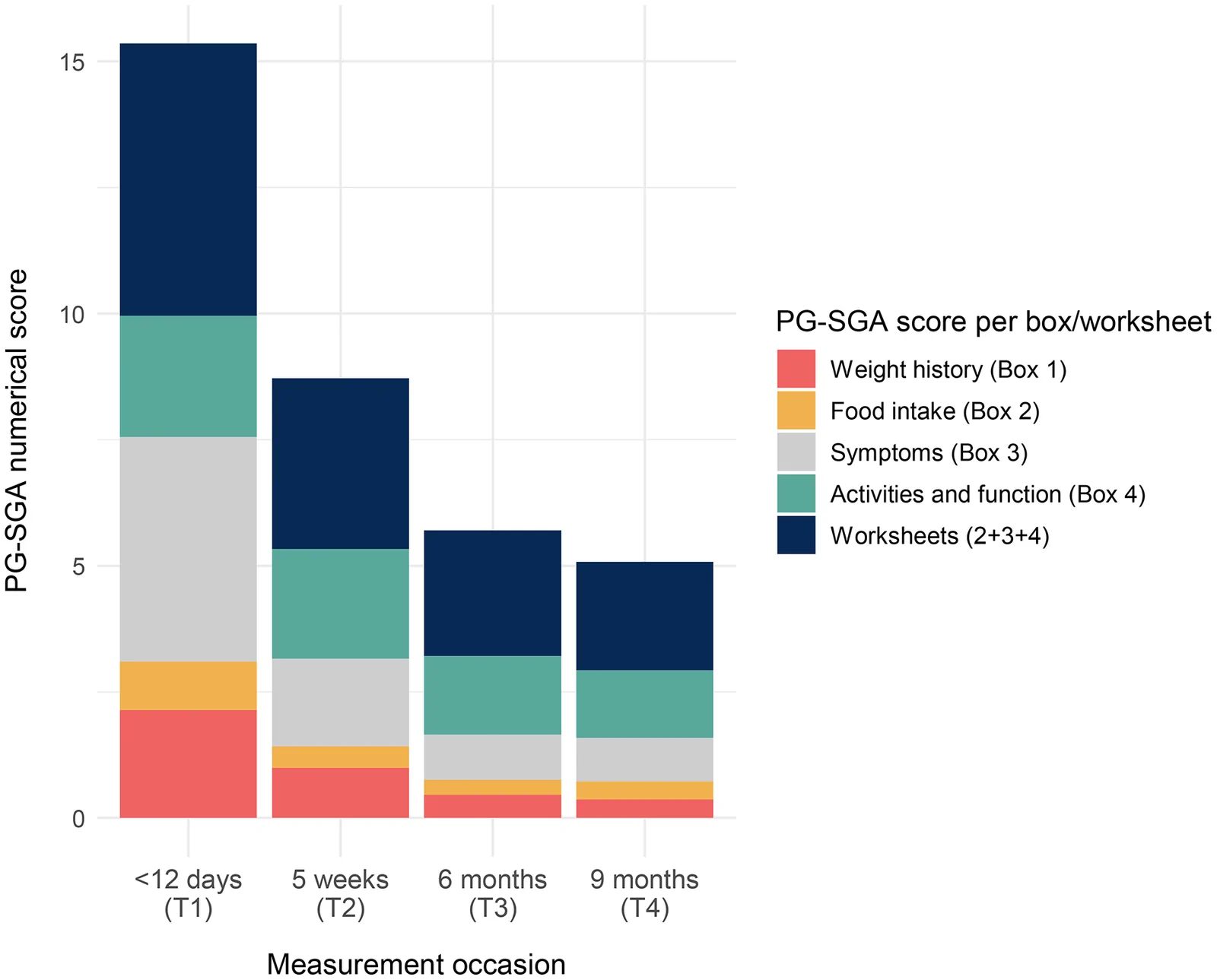
Stacked bar plot of the distribution of average PG-SGA numerical scores at various measurement occasions post-amputation. Abbreviations. PG-SGA, Patient-Generated Subjective Global Assessment. The vertical size corresponds to the mean PG-SGA score over the whole sample per measurement occasion.

Mean grip strength and muscle mass did not significantly differ across the measurement occasions (p = 0.071, p = 0.494). Grip strength increased 2 kg between T1 and T4 (p = 0.060). LLA adjusted body weight and BMI significantly differed over measurement occasions (p = 0.043, p = 0.048), while actual body weight did not (p = 0.052). Actual and LLA adjusted body weight increased 1.9 and 2.1 kg between T2 and T3 (p = 0.086, p = 0.077) and 2.1 and 2.3 kg between T2 and T4 (p = 0.069, p = 0.055). BMI increased 0.7 kg/m^2^ between T2 and T3 (p = 0.077) and 0.7 kg/m^2^ between T2 and T4 (p = 0.064, Table 4, Fig 4).

**Table 4:** Grip strength, muscle mass, body weight, and body mass index at various measurement occasions post-LLA as mean and standard deviation.

		T1 <12 days	T2 5 weeks	T3 6 months	T4 9 months	p-value^a^	p-value^b^
Grip strength (kg)		n = 74	n = 71	n = 57	n = 54		
	Mean	29 ± 11	30 ± 11	31 ± 12	33 ± 10	0.018^¥#^	0.071
	Low (%)^c^	43%	37%	28%	25%		
Muscle mass		n = 68	n = 68	n = 54	n = 52		
	Mean	234 ± 30	234 ± 32	235 ± 37	235 ± 39	0.763	0.494
Body weight (kg)^d^		n = 75	n = 70	n = 54	n = 48		
	Actual	80.1 ± 19.1	78.0 ± 18.0	81.3 ± 19.6	82.4 ± 18.5	0.024^¥^	0.052
	Adjusted for LLA	84.9 ± 19.6	82.7 ± 18.8	85.9 ± 20.6	87.0 ± 19.4	0.021^¥^	0.043
BMI (kg/m^2^)^e^		n = 75	n = 70	n = 54	n = 48		
	Mean	27.0 ± 5.3	26.5 ± 5.2	27.4 ± 5.6	27.5 ± 5.2	0.025^¥^	0.048
	Low (%)	13%	20%	11%	8%		
	Overweight (%)	36%	36%	30%	42%		
	Obesity (%)	28%	24%	31%	27%		

Abbreviations: kg, kilogram; LLA, lower limb amputation; m, meter; BMI, body mass index.

a  = Analysed by mixed effect model, incorporating time.

b  = Analysed by mixed effect model, incorporating time, age, sex, amputation level (low, high), education level (low, mod/high), CCI score without age, smoker (yes, previous/never), and physical activity in hours per week.

c  = Grip strength was categorized as reduced grip strength if <30 kg for males and <20 kg for females [37].

d  = Body weight was measured by a researcher or healthcare professional, with the exception of self-reported weight for 0, 2, 5, and 21 participants at T1, T2, T3, and T4, respectively.

e  = BMI included body weight corrected for LLA. BMI was categorized as low if <20 kg/m^2^ for those younger than 70 years old or <22 kg/m^2^ for those 70 years old or older [38]. Additionally, a BMI of 25–29.9 kg/m^2^ was categorized as overweight and a BMI ≥ 30 kg/m^2^ was classified as obese.

† Significant difference between T1 and T2 (p < 0.05).

§ Significant difference between T1 and T3 (p < 0.05).

# Significant difference between T1 and T4 (p < 0.05).

‡ Significant difference between T2 and T3 (p < 0.05).

¥ Significant difference between T2 and T4 (p < 0.05).

¶ Significant difference between T3 and T4 (p < 0.05).

Cook’s distance ranged from 0.06 to 0.08 for grip strength, 0.11 to 0.15 for muscle mass, 0.05 to 0.11 for body weight and BMI.

**Fig 4:**
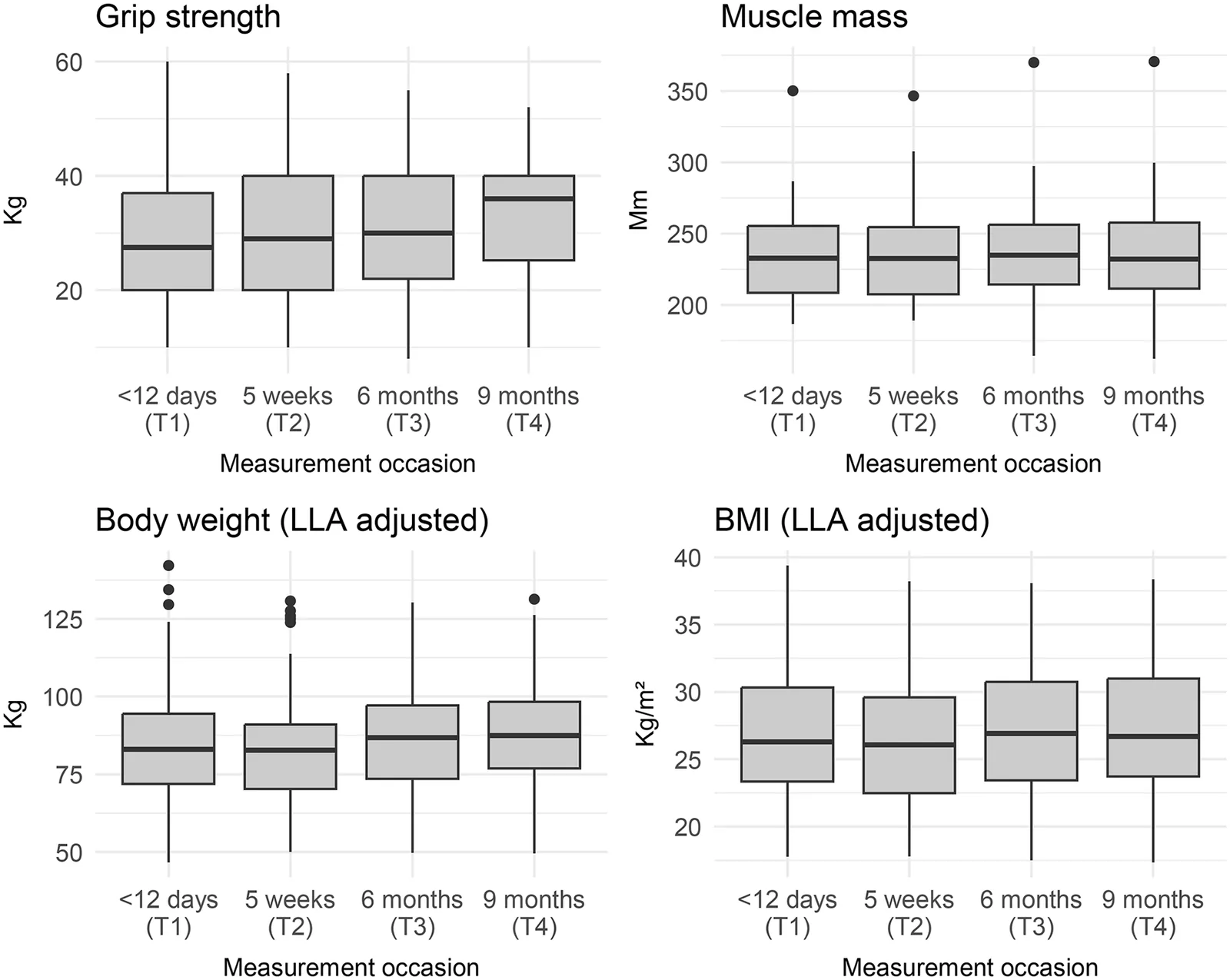
Box-and-whiskers plots of median grip strength, muscle mass, LLA adjusted body weight, and body mass index at various measurement occasions post-amputation. Abbreviations: LLA, lower limb amputation; BMI, body mass index; Kg, kilogram; Mm, millimeter; m, meter. Outlier, defined as value outside 1.5 times the interquartile range.

### Determinants of undernutrition

Results of the univariate analyses to evaluate determinants of undernutrition are presented in Table 5. Significant determinants of undernutrition included: living situation (with individuals living in a care facility being more often undernourished, OR = 6.2, p < 0.001), and physical activity level (with lower physically active people being having a higher prevalence of undernutrition, OR = 0.96 for every hour of physical activity/week, p = 0.006). Sex (females having a higher prevalence of undernutrition, OR = 2.6, p = 0.060) and CCI without incorporating age (OR = 1.3 for every point CCI increase, p = 0.067) were not significant determinants of undernutrition.

**Table 5:** Determinants of undernutrition, based on the category of PG-SGA dichotomized in univariate analysis, incorporating time.

	Odds Ratio	95% confidence interval	β	Standard error	p-value^a^
Intercept	7.81	3.44, 17.73	2.06	0.42	<0.001
Time T1-T2	0.19	0.07, 0.46	−1.69	0.47	<0.001
Time T1-T3	0.04	0.01, 0.11	−3.30	0.57	<0.001
Time T1-T4	0.03	0.01, 0.09	−3.57	0.60	<0.001
Age	1.02	0.98, 1.07	0.02	0.02	0.266
Sex (male ref)	2.57	0.96, 6.87	0.94	0.50	0.060
CCI^b^	1.25	0.98, 1.59	0.22	0.12	0.067
LLA level (low ref)	1.57	0.57, 4.32	0.45	0.52	0.386
Uni/bilat LLA (uni ref)	1.41	0.44, 4.52	0.34	0.59	0.562
Education (low ref)	1.01	0.42, 2.47	0.01	0.45	0.977
Living (home ref)	6.20	2.28, 16.87	1.83	0.51	<0.001
Smoker (current ref)	0.67	0.26, 1.75	−0.40	0.49	0.417
Pain	1.09	0.96, 1.23	0.08	0.06	0.201
Physical activity (hwk)	0.96	0.93, 0.99	−0.04	0.02	0.006

Abbreviations: ref, reference group; CCI, Charlson Comorbidity Index; LLA, lower limb amputation; uni, unilateral; bilat, bilateral; hwk, hours per week.

a  = Analysed by mixed effect model, incorporating time.

b  = Charlson Comorbidity Index without scoring for age, to avoid overlap with parameter age.

Living – all participants were living in a care facility at T1 (not at home).

Smoking status at moment of LLA. Current vs never/previous.

Pain includes VAS on average in the last week.

Physical activity is based on adapted-SQUASH. Physical activity is in hours per week, instead of minutes per week, for easier interpretation.

PG-SGA dichotomized (well-nourished (ref) vs moderate/suspected undernutrition and severely undernourished).

## Discussion

This study aimed to longitudinally determine the nutritional intake and prevalence of undernutrition, as well as its determinants in people with a dysvascular major LLA. Prevalence of inadequate nutritional intake post-LLA is high. Furthermore, the prevalence of undernutrition a few days and five weeks post-LLA is alarmingly high (respectively 83% and 56%), which gradually decreases but remains high long-term post-LLA (28% and 24% at six and nine months post-LLA, respectively). People who have undergone LLA frequently report weight loss, less food intake, nutrition impact symptoms, and limitations in activities/functioning, and have low grip strength and muscle mass. Determinants of undernutrition are living in a care facility and being less physically active.

Our findings align with two previous studies in people with transtibial amputations and those with various major LLAs. Those studies reported an undernutrition prevalence of 72% and 88% during hospital admission [47,48]. Those two studies included multiple domains of undernutrition in their assessments, sharing similarities with our undernutrition assessment. Additionally, a recent study at six months post-minor and -major LLA reported that 57% of people was at risk for undernutrition and 5% was undernourished based on the Mini Nutritional Assessment [49]. To the best of our knowledge, our study is the first study that assessed undernutrition longitudinally in the LLA population. Not only improvement and stability, but also deterioration of the stage of undernutrition were observed over time among the participants. This high prevalence of undernutrition in the LLA population is worrisome, as undernutrition leads to higher mortality rates, impaired wound healing, increased length of hospital stay, higher risk of readmission, lower functional functioning, and reduced quality of life in other various medical populations [38,50–52].

This study provides evidence for a critical need for interdisciplinary (nutritional) intervention following dysvascular major LLA as weight loss, low nutritional intake, nutrition impact symptoms, low functional status, high disease state and metabolic stress and low/loss of muscle mass were highly prevalent. To the best of our knowledge, no evidence-based strategies for nutritional rehabilitation in the LLA population have been published, suggesting that these do not exist. Future interventions should not only aim to ensure an adequate nutritional intake, but also address factors that negatively impact food intake. This is important given the high prevalence of nutrition impact symptoms at every measurement occasion post-LLA, e.g., lack of appetite, nausea, vomiting, and early satiation. Additionally, nutritional counselling is recommended to enhance people’s understanding of adequate nutrition, given the nutritional knowledge that is assumed limited in the LLA population [13,14,53]. Nutritional counselling can empower individuals to make informed nutritional choices that can positively impact their health and recovery [54,55], even when the food provided is not to their taste or personal preference. Moreover, this study highlights a high percentage of body weight loss in the month(s) preceding LLA, with many participants exhibiting low muscle mass and strength. Therefore, initiating nutritional interventions earlier, for patients at high risk of LLA, may help prevent or limit undernutrition. Coordinated care across hospital settings, rehabilitation or skilled nursing facilities, and during the transition to home may be beneficial for the continuity and effectiveness of nutritional support.

Our study revealed different determinants of undernutrition. In line with earlier research in the elderly population, we found that people living in care facilities have a higher risk of undernutrition [56,57]. This finding may be counterintuitive, as healthcare professionals are expected to actively monitor and manage nutritional status. However, time constraints, lack of knowledge about nutrition, and gaps in training and education regarding the importance of nutrition and how to address it effectively may contribute to inadequate nutritional care [53,58–61]. Moreover, residents in care facilities may have limited control over their diet, possibly leading to dissatisfaction with meal options and potentially lower intake. Additionally, residents in care facilities often have poorer physical health, further exacerbating their vulnerability to undernutrition. Furthermore, we found that lower levels of physical activity were associated with a higher risk of undernutrition, in line with earlier research in the elderly population [57]. By being less physically active the anabolic stimulus is reduced, which may lead to muscle loss [62], underscoring the importance of maintaining an appropriate level of physical activity to support nutritional status and overall health. Moreover, having more severe comorbidities showed a clinically relevant—but not statistically significant—association with undernutrition. A poorer health status may result in increased nutritional requirements, while it simultaneously may decrease food intake due to nutrition-impact symptoms such as loss of appetite and gastrointestinal issues. Lastly, females had a non-significant trend of higher prevalence of undernutrition, which is consistent with previous research on risk of undernutrition in vascular surgery patients [63]. Previous research in people with peripheral artery disease showed that females have lower physical functioning, higher risk for frailty and depression, more complications and higher mortality rate compared to males with peripheral artery disease [64–66], which may explain the possible sex differences that we found in our study. However, it is important to note the small sample size of females (n = 22), which may limit the robustness of these findings. Although identifying these determinants of undernutrition is valuable, the high prevalence of undernutrition in the acute post LLA phase underscores that all patients, regardless of the presence or absence of these determinants, should undergo nutritional assessment and diagnostics, receive appropriate dietary counselling and support, and be monitored regularly to prevent under recognition and delayed nutritional intervention.

Our study indicates that inadequate nutritional intake and poor diet quality are highly prevalent in the dysvascular major LLA population. Adequate nutritional intake is important for wound healing, preventing further health complications, and supporting overall recovery [19]. Moreover, the diet quality observed in our study is somewhat lower than that of the general Dutch population [26], as indicated by the lower scores on the Dutch Healthy Diet index of the participants. A low diet quality is concerning because the Health Council of the Netherlands derived the Dutch dietary guidelines [25] to reduce the risk of the top ten major chronic diseases, underscoring the potential health risks. It is important to note that during periods of illness or recovery, such as the weeks following dysvascular LLA, and when undernourished, protein requirements are increased. In the phase of illness and recovery, or when undernourished, the nutritional interventions’ primary focus should be on ensuring adequate energy and protein intake to optimize recovery. This may involve prioritizing immediate recovery needs, even if they temporarily differ from the recommendations for long-term health, such as diet quality.

A strength of this study is the multiple follow-ups and the relatively large sample size for a study in people who have undergone a dysvascular major LLA. However, for statistical analyses, this sample size may still be considered relatively small, whereby we were constrained to dichotomize undernutrition and potential determinants (LLA level, education, living situation, smoking status). Furthermore, the study captures a snapshot in time, and nutritional intake and nutritional status may vary over time within people, which could have affected the results. Body weight measurements were performed using various scales, which introduced variability due to rounding and scale limitations, especially notable at T3 and T4, but also present at T1 and T2 depending on the scale of the hospital or rehabilitation facility if weighing on a personal scale was not feasible. General alcohol use was not assessed, as we believed self-reported results would be unreliable. Alcohol use was questioned in the 24-hour recall. Furthermore, smoking status was only assessed at the time of LLA. We expect limited effect on the results, as most pre-LLA smokers likely continued smoking one year post-LLA [67].

In this study, we encountered several methodological challenges in determining the prevalence of undernutrition. Firstly, at T1, the body weight post-LLA, corrected for the missing limb(s), was compared to the self-reported body weight before LLA. The correction formula for the weight of the amputated limb is not validated and may vary on an individual level due to differences in residual limb lengths, morphology, and body composition, including atrophy in the residual limb [68]. However, other parameters of undernutrition make the reported high prevalence of undernutrition at T1 likely, as 69% of participants reported weight loss in the last two weeks, 62% reported eating less, and many participants had low muscle mass or muscle loss. Secondly, fluid retention can lead to an underestimation of undernutrition at T1, as the excess fluid may mask clinically relevant weight loss. At T2, fluid loss can result in an overestimation of undernutrition, as the reduction in body weight due to fluid loss may be mistakenly attributed to undernutrition rather than the loss of retained fluid. Thirdly, when weighing was not feasible, a score of zero in the PG-SGA was assigned, potentially leading to underreporting of (the stage of) undernutrition. Fourthly, participants may have underestimated the extent of their reduced food intake in Box 2 of the PG-SGA, as suggested by discrepancies with their 24-hour recall data, potentially indicating even worse stages of undernutrition than reported. Fifthly, selection bias may have occurred, as patients who refused to participate due to physical or psychological problems could be at higher risk for undernutrition, indicating an even worse prevalence of undernutrition. Sixthly, it is important to acknowledge that our findings on nutritional intake are based on a single 24-hour recall, which may limit validity on individual level due to daily variations in intake. In the dysvascular LLA population, additional reliability challenges exist, including memory loss, unfamiliarity with food products or preparation, and inaccurate reporting [69,70]. Nevertheless, the 24-hour recall was the best of the feasible methods to use in this study. For example, it is suggested that a 3-day food diary is not feasible for the majority of people undergoing dysvascular LLA due to memory problems, and a food frequency questionnaire assesses usual intake, which likely differs in the acute phase post-LLA and limits the ability to detect changes in nutritional intake over time. Additionally, little is known about the nutritional requirements for people with a non-intact body. Therefore, more accurate methods are needed to evaluate nutritional intake and requirements in the LLA population. Similarly, although BMI was calculated using corrected body weight to account for the missing limb, this method relies on a generalized formula that may not reflect individual differences post-LLA, and its validity in the LLA population remains uncertain. Seventhly, the underlying vascular etiology of amputation was not further specified, which limited subgroup analyses to identify potential differences in nutritional risk profiles across vascular conditions. Lastly, participants were included in the Northern Netherlands, which may limit generalizability to people in other Dutch regions and countries with different diets.

Future research should investigate the association between undernutrition and clinical outcomes in the dysvascular LLA population, as well as the effectiveness of interventions aimed at improving nutritional status and, consequently, clinical outcomes. These future interventions should be tailored to persons with a dysvascular LLA, for instance regarding health literacy [71–73]. Furthermore, since physical activity was identified as a determinant of undernutrition, future research should evaluate physical activity levels pre-, peri-, and post-dysvascular LLA, to identify opportunities to optimize physical activity levels, for anabolic benefits and health outcomes.

## Conclusion

Inadequate nutritional intake and undernutrition are alarmingly prevalent post dysvascular LLA. Prevalence of undernutrition is highest during the acute phase following amputation. While the prevalence decreases over time, it remains high at about one over four persons at six and nine months post-amputation. People who have undergone a dysvascular major LLA frequently report weight loss, less food intake, nutrition impact symptoms and limitations in activities/functioning, as well as having low grip strength and muscle mass. Determinants of undernutrition include living in a care facility and lower physical activity. Timely interventions are highly needed to improve nutritional status, to potentially optimize clinical outcomes in the dysvascular major LLA population.
